# Supplementary figures and images for: The role of the AP-1 adaptor complex in outgoing and incoming membrane traffic
Source: J Cell Biol. 2024 Apr 5;223(7):e202310071. doi: 10.1083/jcb.202310071 (PMC10996651; doi:10.1083/jcb.202310071)

B

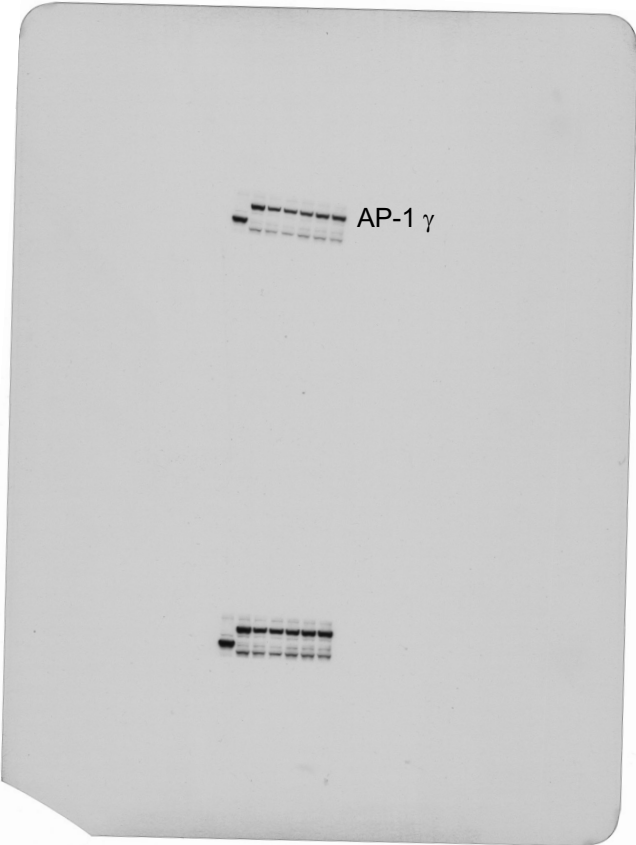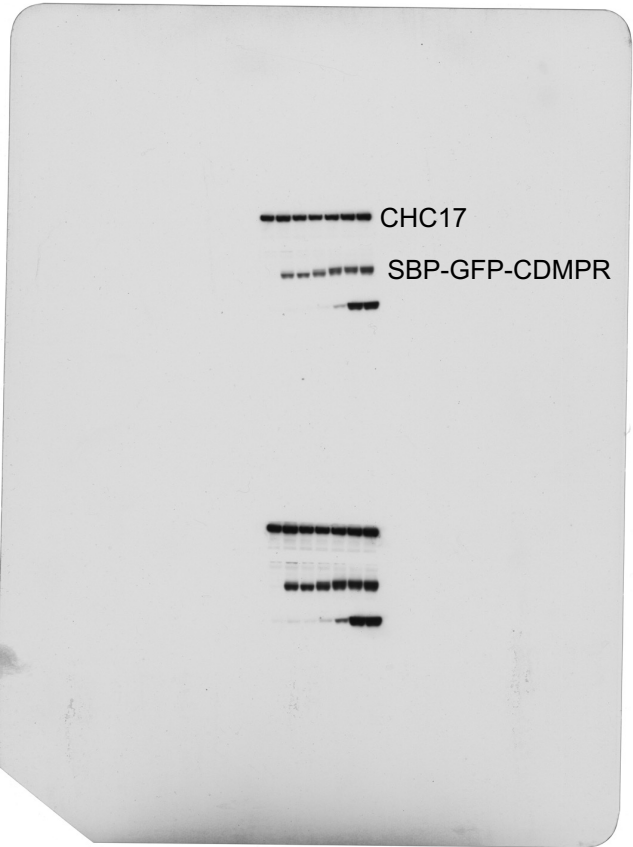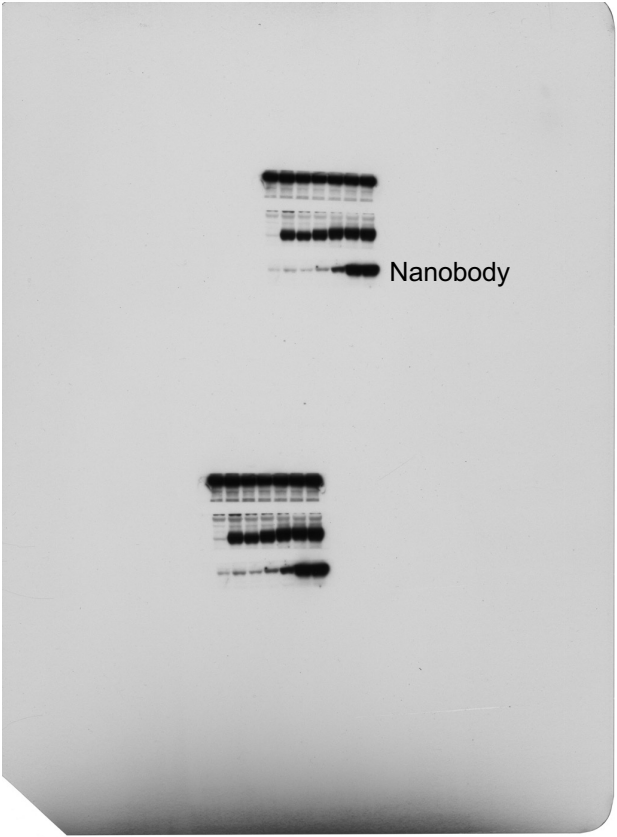

Supplement: SourceData F2 — is the source file for Fig. 2. [file JCB_202310071_SourceDataF2.pdf]

C

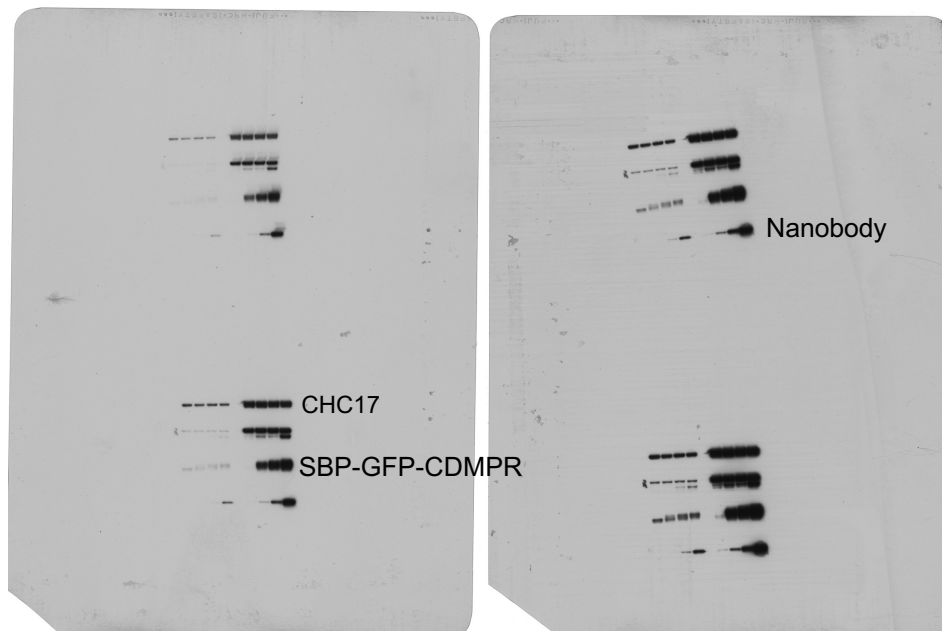

E

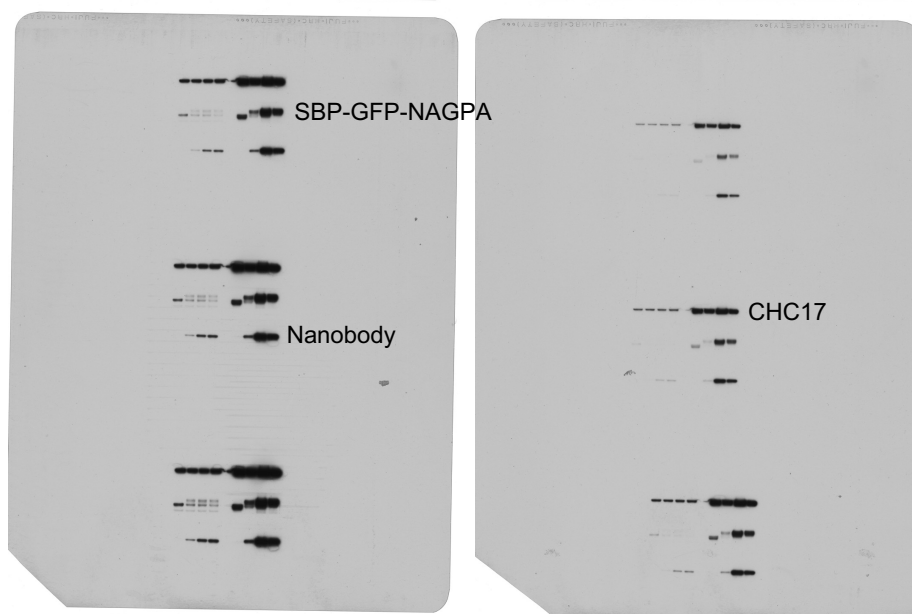

G

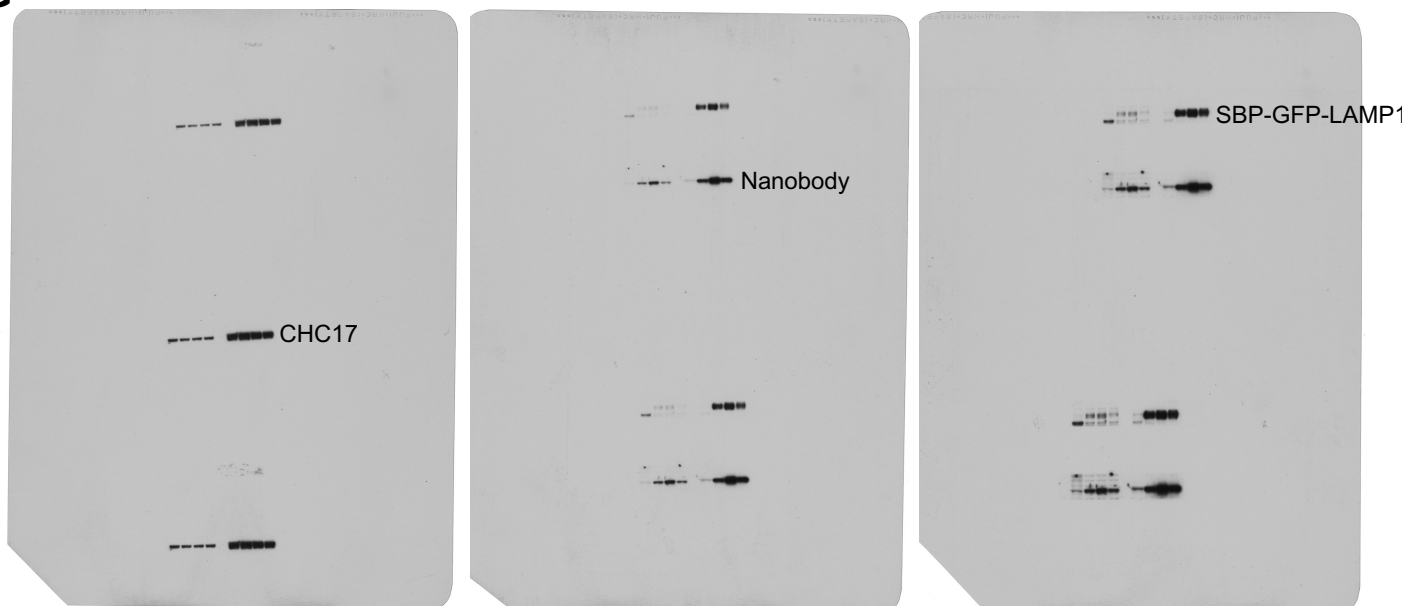

Supplement: SourceData F5 — is the source file for Fig. 5. [file JCB_202310071_SourceDataF5.pdf]

**B**

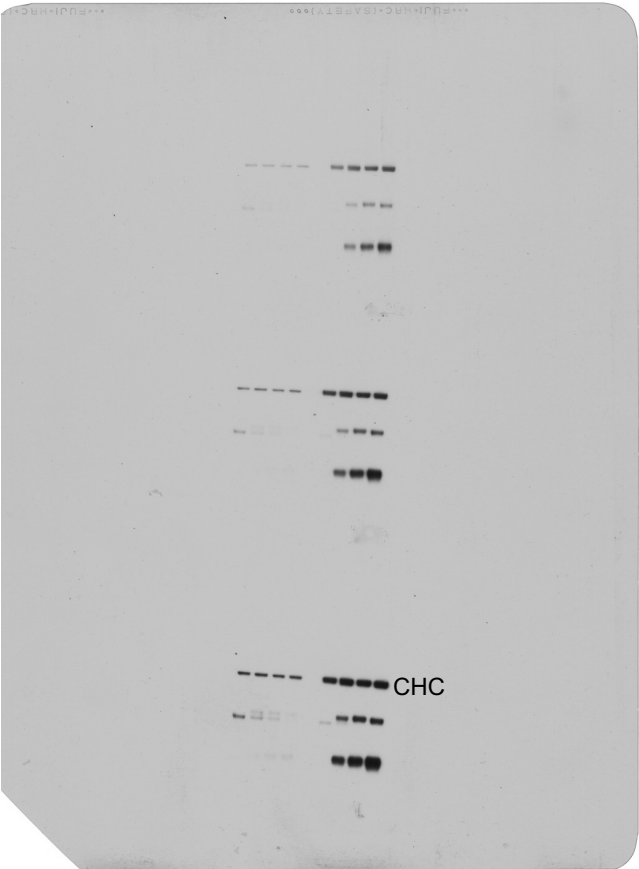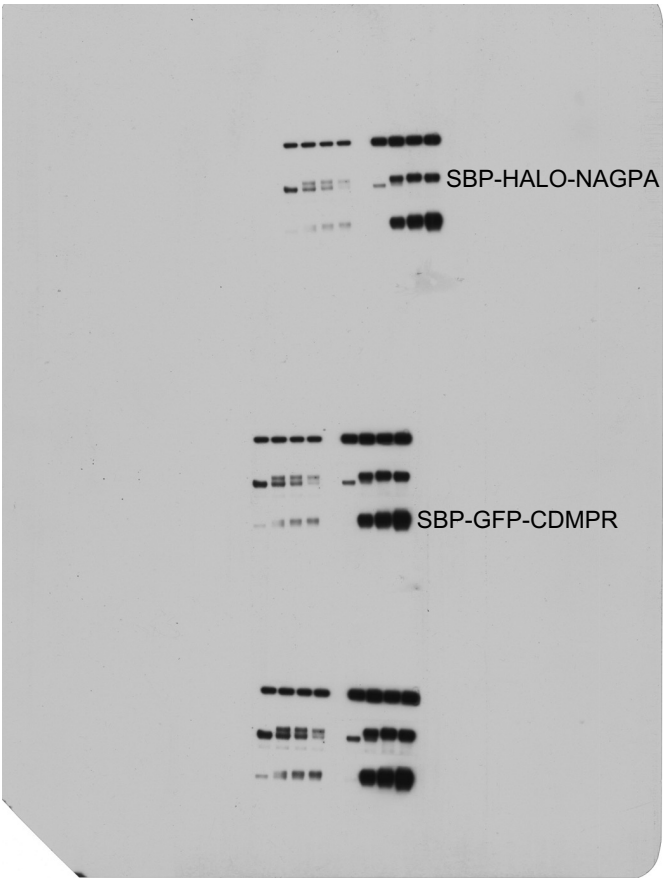

Supplement: SourceData F6 — is the source file for Fig. 6. [file JCB_202310071_SourceDataF6.pdf]

**B**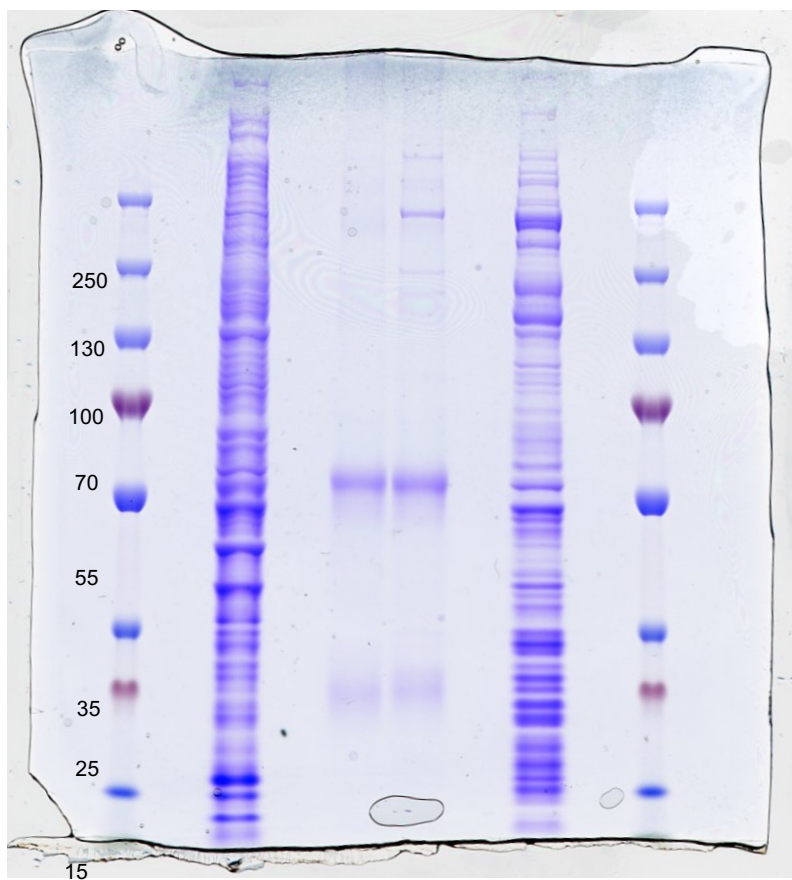**C**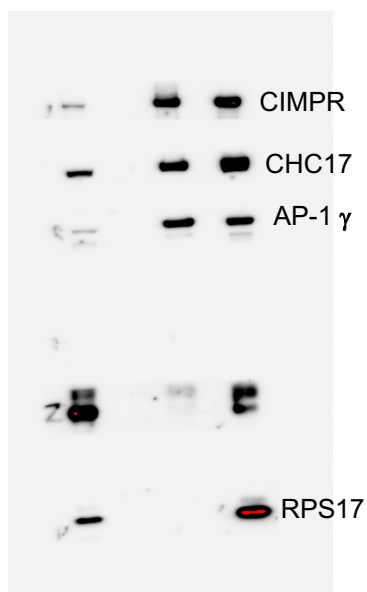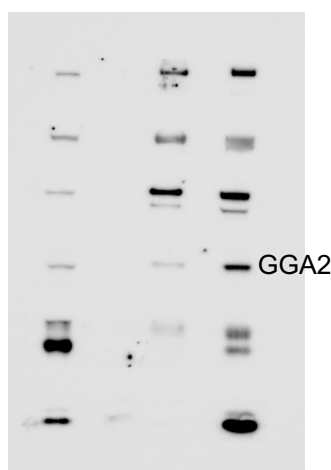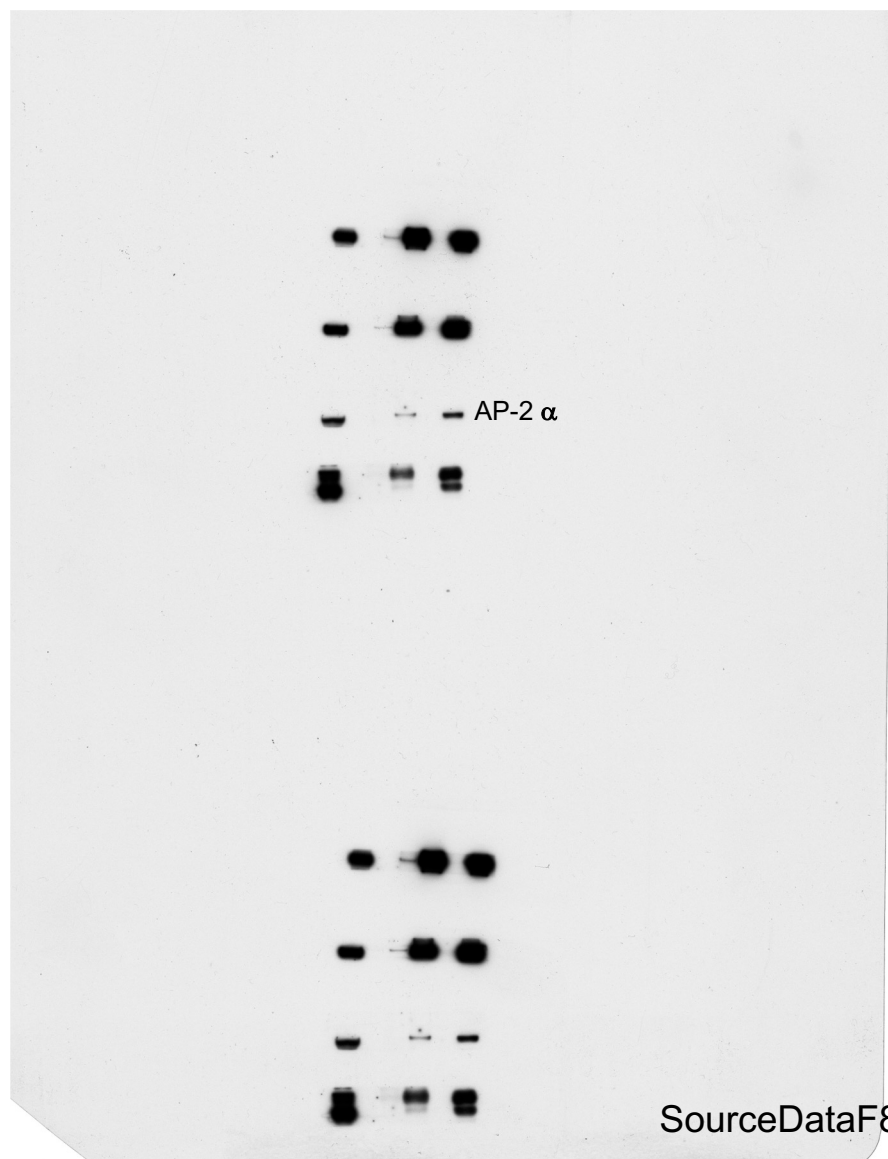

Supplement: SourceData F8 — is the source file for Fig. 8. [file JCB_202310071_SourceDataF8.pdf]

**A**

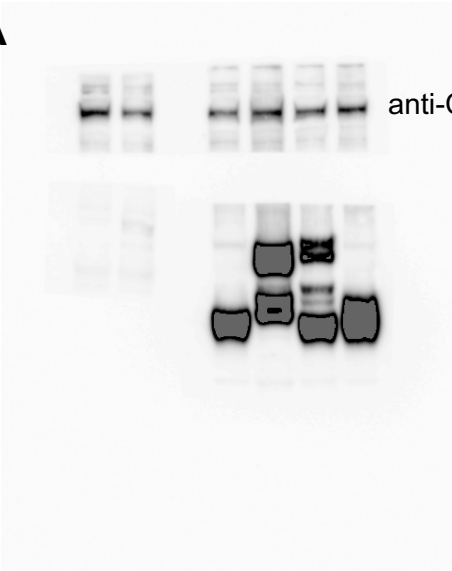

anti-CIMPR

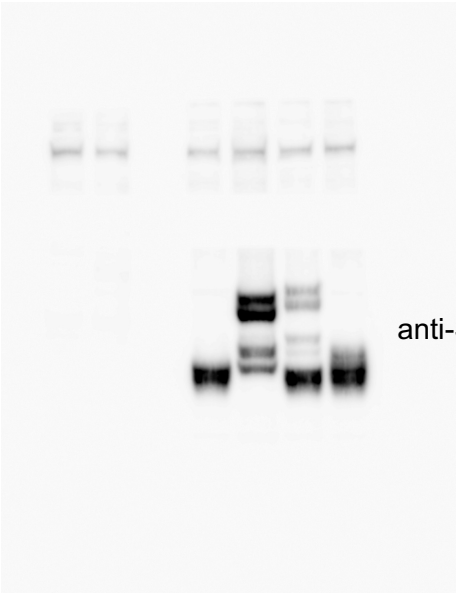

anti-SBP

Supplement: SourceData FS4 — is the source file for Fig. S4. [file JCB_202310071_SourceDataFS4.pdf]
