# Supplementary material for: The role of the AP-1 adaptor complex in outgoing and incoming membrane traffic
Source: J Cell Biol. 2024 Apr 5;223(7):e202310071. doi: 10.1083/jcb.202310071 (PMC10996651; doi:10.1083/jcb.202310071)
Supplement: SourceData FS1 — is the source file for Fig. S1. [file JCB_202310071_SourceDataFS1.pdf]

A

First 10 lanes
in the figure

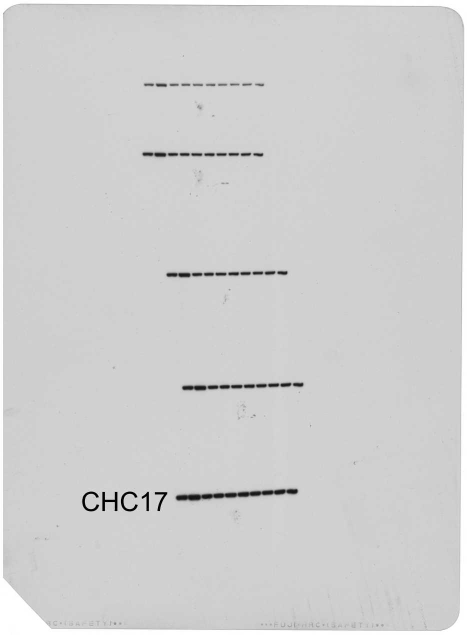

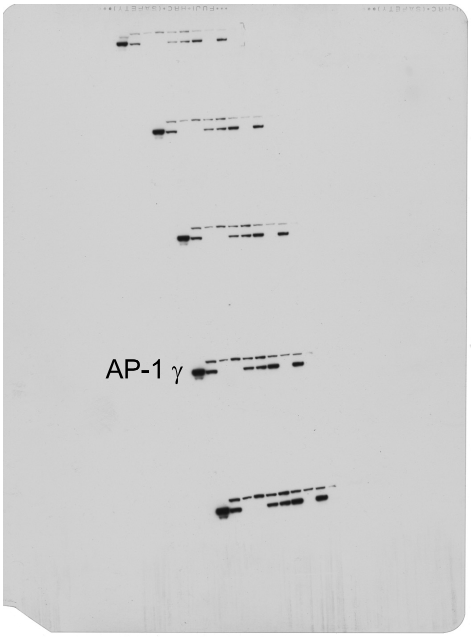

Middle 6 lanes
(1st 2 lanes on the left not used)

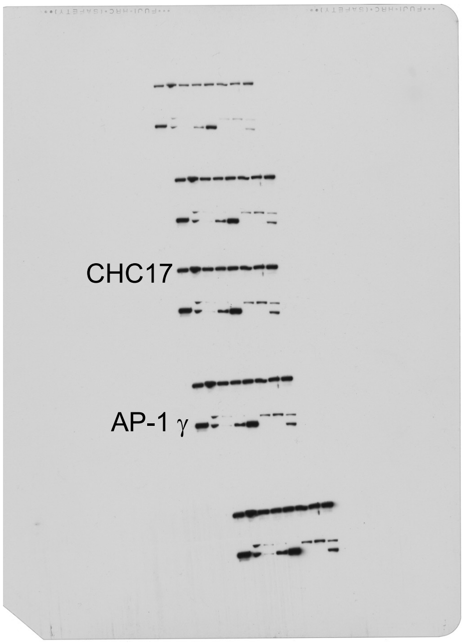

Last 3 lanes
(lanes 2-4 on the blot)

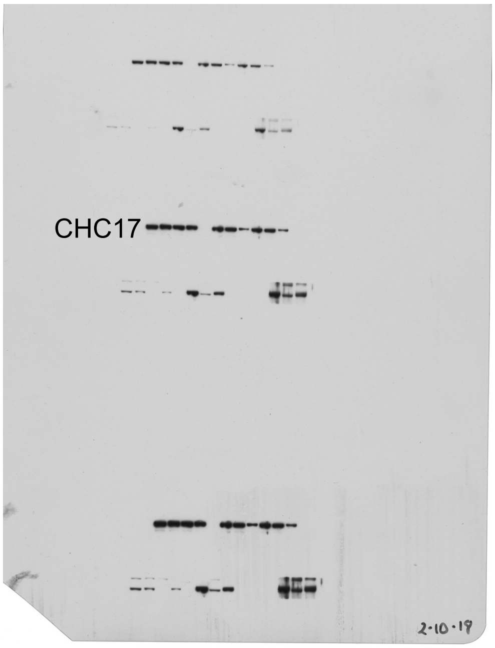

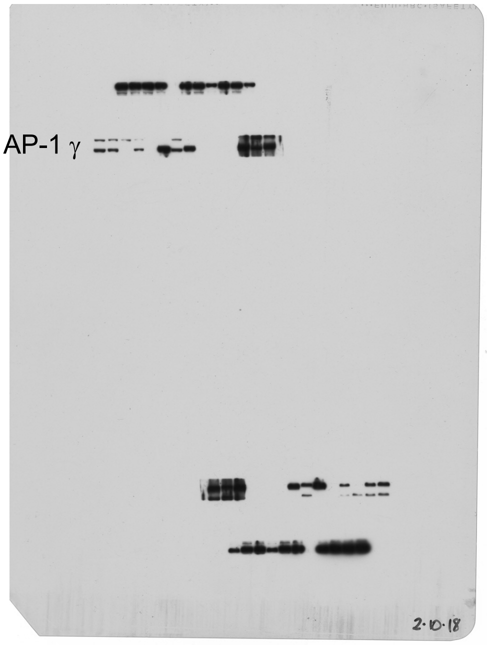

SourceDataFS1
